# Corticosteroid-Binding Globulin: Structure-Function Implications from Species Differences

**DOI:** 10.1371/journal.pone.0052759

**Published:** 2012-12-26

**Authors:** Bernd R. Gardill, Michael R. Vogl, Hai-Yan Lin, Geoffrey L. Hammond, Yves A. Muller

**Affiliations:** 1 Department of Biology, Lehrstuhl für Biotechnik, Friedrich-Alexander-University Erlangen-Nuremberg, Erlangen, Germany; 2 State Key Laboratory of Reproductive Biology, Institute of Zoology, Chinese Academy of Sciences, Beijing, China; 3 Department of Obstetrics and Gynecology, Child and Family Research Institute, University of British Columbia, Vancouver, British Columbia, Canada; Griffith University, Australia

## Abstract

Corticosteroid-binding globulin (CBG) transports glucocorticoids and progesterone in the blood and thereby modulates the tissue availability of these hormones. As a member of the serine protease inhibitor (SERPIN) family, CBG displays a reactive center loop (RCL) that is targeted by proteinases. Cleavage of the RCL is thought to trigger a SERPIN-typical stressed-to-relaxed (S-to-R) transition that leads to marked structural rearrangements and a reduced steroid-binding affinity. To characterize structure-function relationships in CBG we studied various conformational states of *E. coli*-produced rat and human CBG. In the 2.5 Å crystal structure of human CBG in complex with progesterone, the RCL is cleaved at a novel site that differs from the known human neutrophil elastase recognition site. Although the cleaved RCL segment is five residues longer than anticipated, it becomes an integral part of β-sheet A as a result of the S-to-R transition. The atomic interactions observed between progesterone and CBG explain the lower affinity of progesterone in comparison to corticosteroids. Surprisingly, CD measurements in combination with thermal unfolding experiments show that rat CBG fails to undergo an S-to-R transition upon proteolytic cleavage of the RCL hinting that the S-to-R transition observed in human CBG is not a prerequisite for CBG function in rat. This observation cautions against drawing general conclusions about molecular mechanisms by comparing and merging structural data from different species.

## Introduction

Corticosteroid-binding globulin (CBG) is a glycosylated plasma protein implicated in steroid transport and delivery [1]. In human CBG, the peptide chain consists of 383 amino acids and includes six sites for N-glycosylation that are differentially utilized [2], [3]. By its primary structure, CBG is classified as a clade A member of the serine protease inhibitor (SERPIN) family, together with the closely related alpha-1-antichymotrypsin, alpha-1-antitrypsin (AAT) and thyroxine-binding globulin (TBG) [2], [4]. Contrary to the proteinase inhibitors alpha-1-antichymotrypsin and AAT, CBG and TBG have no known inhibitory function. By contrast, they function as transport proteins for small lipophilic hormones, i.e. steroids and thyroid hormone, respectively [2], [5].

The importance of CBG is highlighted by its ability to bind 80–90% of cortisol in plasma, leaving only about 4–5% circulating in the free fraction and the remainder bound loosely to albumin [6]. Several natural occurring CBG variants have been identified that are either not produced appropriately [7], [8], [9] or have steroid-binding defects [10], [11], [12]. In humans with CBG deficiencies symptoms of hypotension and chronic pain have been reported although the pathological basis of these clinical conditions remain unclear [8], [10].

Interestingly CBG is a ‘negative acute-phase protein’ during inflammatory response, and its plasma concentration decreases rapidly during sepsis [13], severe burn [14] and myocardial infarction [15], and this likely increases the amounts of free glucocorticoids that can control the inflammatory response, gluconeogenesis, and stress. While CBG does not act as a protease inhibitor, it is a substrate for neutrophil elastase and becomes cleaved in the reactive center loop (RCL) [5]. In most SERPINs, cleavage of the RCL triggers an S-to-R transition during which the N-terminal part of the RCL inserts into β-sheet A to make it fully antiparallel [16]. After the discovery that CBG is a SERPIN family member [2], this conformational transition was proposed as an important mechanism for steroid release at sites of inflammation [5], [11].

Recently, crystal structures of rat CBG [17], a cleaved human CBG-AAT chimera [18] and TBG [19] were solved, and these structures suggested an allosteric coupling mechanism that links the RCL conformation to the changes in ligand-binding affinity. In this model, helix D has been suggested as a connecting element that functions by partial unwinding upon RCL insertion. By its position between β-sheet A and β-sheet B, the RCL can connect these important structural entities and regulate ligand binding and release in a manner similar to the modulation of heparin binding to antithrombin [18], [20].

The observation that the steroid-binding affinity in human CBG decreases 16-fold upon a temperature increase from 35 to 42°C [21] led to the proposal that this constitutes a mechanism to accentuate the free concentrations of cortisol in the blood during fever or locally at sites of increased body temperature [21], [22]. This is possibly accomplished in CBG by a “flip-flop” mechanism. In the absence of ligand, the C-terminal part of the RCL partially inserts in between strands 3 and 5 of β-sheet A, whereas the RCL becomes fully expelled from β-sheet A when the ligand-binding site is occupied [17], [18], [19]. This mechanism could make use of the same allosteric coupling pathway, i.e., the coupling of the RCL insertion with the ligand-binding site *via* helix D, as discussed for the irreversible loss of high affinity binding upon RCL cleavage.

To further define the connection between RCL conformation and steroid-binding affinity we have characterized rat and human CBG variants, and compared their steroid-binding parameters in the context of different conformational states. We describe the first crystal structure of human CBG in complex with progesterone, and data from isothermal titration calorimetry (ITC) and thermal denaturation studies that reveal unexpected differences in the behavior of human and rat CBG after proteolytic cleavage of the RCL loop.

## Materials and Methods

### CBG plasmid constructs and site-directed mutagenesis

The coding sequences of residues 11 to 383 of human CBG (Swiss-Prot entry P08185) and residues 5 to 374 of rat CBG (P31211) were cloned into a pGEX-2T vector (GE Healthcare, Uppsala), yielding N-terminal glutathione-S-transferase (GST) fusion proteins. Mutations were introduced using a two-stage PCR procedure based on the QuikChange (Stratagene, Santa Clara) site-directed mutagenesis protocol [23]. All experiments with human CBG were conducted with a mutant in which surface residue Lys126 was replaced by alanine in order to facilitate crystallization [24]. Wild-type rat CBG (rat CBG-WT) and rat CBG mutants CBG-RCL1, CBG-RCL2 and CBG-RCL3 were produced to probe the function of the RCL residues in rat CBG. Primer pairs used for mutagenesis are listed in Table S1. All gene constructs were confirmed by DNA sequencing prior to protein production.

### Protein production and purification

The GST-tagged human and rat CBG variants were produced in *E. coli* BL21 Star™ (DE3) cells (Invitrogen, Carlsbad) using the ZYM-5052 auto-inducing medium [25]. For each variant, 10 L of ZYM-5052 medium containing 100 µg/mL ampicillin were inoculated with an overnight culture of a single clone. The flasks were incubated at 37°C until the optical density at 600 nm reached 0.3. The temperature was then lowered to 20°C, and the cells were allowed to grow for an additional 20 h. The pellet was resuspended in PBS buffer (pH 7.4) containing 5 mM EDTA after cell harvesting (4 mL of buffer per gram of pellet).

The supernatant obtained after ultrasonification and centrifugation was diluted 4 times with buffer and applied to a Glutathione Sepharose column (GE Healthcare). The fusion protein was eluted in a buffer containing 50 mM Tris-HCl, pH 7.9, 50 mM NaCl and 10 mM reduced glutathione. The GST-tag was cleaved off by overnight incubation at 4°C with 5 units bovine plasma thrombin per mg fusion protein (Sigma-Aldrich, St.Louis). After addition of protease inhibitor, the protein solution was loaded on a 10 mL Q-Sepharose FF column (GE Healthcare) and eluted by a linear salt gradient from 50 mM to 1 M NaCl in the presence of 50 mM Tris-HCl (pH 7.9). GST and CBG were co-eluted at low salt concentrations. Fractions containing a mixture of GST and CBG were pooled and loaded again onto a Glutathione Sepharose column, and the flow through was collected. In a final step, the protein was purified by gel filtration chromatography using a Superdex 75 column (GE Healthcare).

### Crystallization, crystallographic data collection and structure determination

Human CBG was concentrated to 10 mg/mL and set up for sitting drop crystallization trials with a drop size of 0.1 µL. A single crystal of human CBG suitable for structure analysis grew in the presence of progesterone in 0.1 M HEPES, pH 7.5, 2% PEG 400, 2 M ammonium sulfate at 19°C. Diffraction data were collected at 100 K at synchrotron beam line BESSY-MX 14.1 at Helmholtz-Zentrum Berlin to a resolution of 2.5 Å. The crystal was flash frozen in liquid nitrogen in a cryosolution containing 80% reservoir solution and 20% ethylene glycol. The diffraction pattern significantly improved after a 2 sec and 4 sec crystal annealing steps. Data were processed using XDS and scaled with XSCALE [26].

The structure was solved *via* molecular replacement with program Phaser [27]. The search model consisted of a slightly modified version of the Protein Data Bank entry 2VDY [18]. The model was manually altered so that the RCL sequence corresponded to that of human CBG and not of AAT as present in the deposited human CBG-AAT (Pittsburgh variant) chimera structure [18]. *R_work_* decreased to 23.0% and *R_free_* to 28.6% during a first automated refinement round with program REFMAC5 [28]. The structure was refined further while alternating between rounds of manual model building in COOT [29] and automated restrained atom position and individual B-factor refinement. 10 TLS groups were identified with the help of the TLSMD web server and included into the model refinement with REFMAC5 during the final stages of refinement [30], [31]. The resulting final TLS tensors were analyzed using TLSANL [32]. Crystallographic data collection and refinement statistics are summarized in Table 1. Structural alignments and calculations of root mean square deviation (RMSD) were done using LSQKAB within the CCP4 program suite [33]. All structure illustrations were created with PYMOL [34].

**Table 1:** Crystallographic data collection and refinement statistics.

Data collection	
X-ray source	BL14.1 BESSY-MX Berlin
Wavelength	0.9184 Å
Detector	Mar CCD MX-225
Spacegroup	C222_1_
Unit cell parameters(Å)	a = 89.2, b = 122.7,c = 81.9
Matthews coefficient (Å^3^ Da^−1^)	2.68
Molecules per asymmetric unit	1
Resolution range (Å)^a^	34 − 2.48 (2.63 - 2.48)
Completeness (%)	99.6 (97.6)
*R*_obs_ (%)^b^	12.2 (59.8)
*R*_meas_ (%)	13.2 (64.6)
Unique reflections	16184 (2514)
Average redundancy	7.2 (7.0)
Average *I/σ(I)*	12.9 (3.2)
Wilson B-factor (Å^2^)	41.6

Refinement statistics	
*R*_work_/*R*_free_/*R*_total_ (%)^c^	18.4/23.9/18.9
R.m.s.d. from ideal geometry	
Bond lengths (Å)	0.009
Bond angles (°)	1.23
Ramachandran plot	
(% of residues in core/additionally allowed regions)^d^	91.7/8.3
Average *B*-values (Å^2^)	
All atoms	34.2
Protein main chain atoms	33.2
Protein side chain atoms	35.5
Ligand atoms	53.0
Solvent atoms	21.4
Cys/Ethylenglycol	44.6/55.8
No. of protein atoms	2878
No. of solvent atoms	111

a Values for the highest resolution shell in parentheses.

b *R*_obs_ = ∑|*I_j_*−<*I*>|/∑ *I_j_* , where *I_j_* is the measured intensity of reflection *j*, and <*I*> is the mean intensity of symmetry-related reflections.

c Ten percent of reflections were randomly assigned to *R*_free_ set.

d Program PROCHECK [50].

### Neutrophil elastase cleavage

Cleavage of the RCL in human and rat CBG mutants was accomplished with 100 mU human neutrophil elastase (Sigma-Aldrich) per mg purified CBG at 30°C and monitored by SDS-PAGE. The cleavage reaction was stopped with AEBSF.

### Circular dichroism (CD) spectroscopy

The purified samples were analyzed in 10 mM potassium phosphate buffer (pH 7.4) with either a Jasco J-815 with PTC-423S or a Jasco J-600A spectropolarimeter equipped with a PTC-348 WI peltier element (Jasco, Tokyo). Far-UV spectra were recorded at 25°C in a 0.1 cm cell with protein concentrations between 1 and 2.5 µM. The data pitch was 0.2 nm and a 1 nm bandwidth with a sensitivity of 10 mdeg and a response time of 1 sec was used. Each spectrum was accumulated either 8 times (20 nm/min) or 12 times (100 nm/min).

Heat-induced unfolding transitions were recorded at protein concentrations between 0.5 and 0.7 µM in 10 mM potassium phosphate (pH 7.4). The samples were heated at a rate of 1°C/min in a 1 cm cell and the ellipticity was monitored at 222 or 220 nm. The sensitivity was set to 50 mdeg with a bandwidth of 1 nm and 8 sec response time [35].

### Isothermal titration calorimetry (ITC)

The protein samples were dialyzed against ITC buffer (20 mM potassium phosphate, 50 mM NaCl, pH 7.4) and titrated with steroids in a VP-ITC microcalorimeter (Microcal, Piscataway). All solutions were filtered (0.2 µm filter pore size) and degassed for 30 min prior to the titration experiments. The sample cell was filled with protein solution containing 15 µM rat CBG or 20 µM human CBG and titrated with steroid solutions containing 150 µM and 200 µM ligand, respectively. The titrations consisted of an initial injection of 2 µL followed by 25 consecutive injections of 10 µL ligand solution at 25°C. A spacing of 240 sec between the injections was used to allow a return to the baseline. Data points from the initial injection, which are generally affected by diffusion during the initial equilibration period after experiment setup, were discarded prior to data analysis. Titration of buffer with ligand solution showed no significant heat of dilution. The binding curve was generated by plotting the heats of injections against the ratio of ligand to protein in the cell. Integrated heat data were analyzed using nonlinear regression fit with a one-site-binding model in the Origin-7™ software provided with the VP-ITC. Changes in the free energy and entropy can be calculated using the equation: Δ*G°* = −*RTlnK* = Δ*H°*−*T*Δ*S°*. In this equation Δ*G°* is the change in Gibbs free energy, R the universal gas constant, T the temperature, and K the association constant. The change in enthalpy is denoted as Δ*H°*, and Δ*S°* is the change in entropy under standard conditions [36]. The data shown in Table 2 were obtained from single titrations of each complex.

**Table 2:** Steroid-binding data.

	*K*_d_ (nM)	*ΔH°*	*TΔS°*	*ΔG°*	*K*_d_ (nM) 37°C
	25°C	(kJ/mol)	(kJ/mol)	(kJ/mol)	[22]
**Hydrocortisone titrated to**					
Rat CBG-RCL1	95±7	−161.6	−121.4	−40.2	
Cleaved rat CBG-RCL1	209±20	−165.8	−127.3	−38.5	
Human CBG-WT	45±7	−151.2	−109.1	−42.1	292.2±19.0
Cleaved human CBG-WT	442±10	−114.7	−78.4	−36.3	1366.0±123.5
Rat CBG-WT	122±6	−124.6	−85.1	−39.5	
**Corticosterone titrated to**					
Rat CBG -WT	37±2	−103.6	−60.9	−42.7	

## Results

### The crystal structure of cleaved human CBG displays the R-conformation and reveals a novel cleavage site

**Figure 1:**
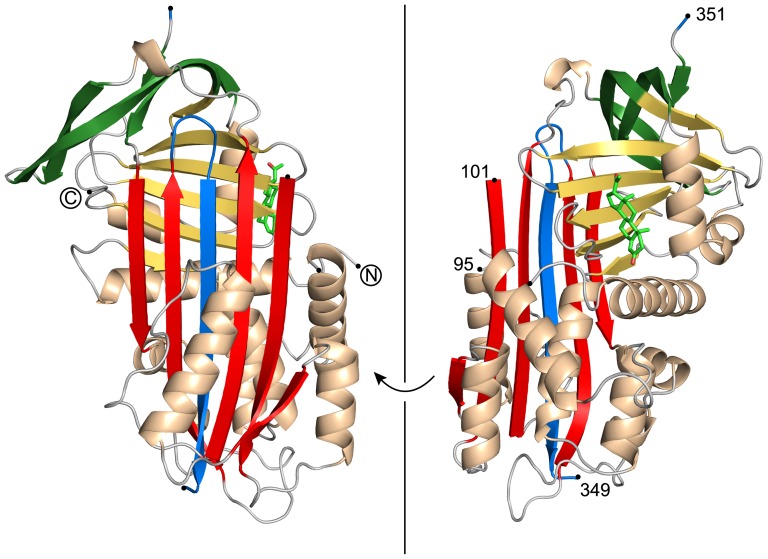
Crystal structure of cleaved human CBG in complex with progesterone. The central β-sheet A is shown in red, with the inserted RCL as part of it in blue. β-sheet C and β-sheet B are colored in green and yellow, respectively. On top of β-sheet B the bound steroid hormone progesterone is depicted in bright green. Electron density for residues 96 to 100 and residue 350 is missing. Chain breaks are shown with black dots.

The crystal structure of human CBG determined here differs from previous human CBG structures in two aspects. It represents the first crystal structure of CBG in complex with progesterone and, more importantly, the sequence of the RCL corresponds to that of wild-type CBG and is not replaced by the sequence of AAT Pittsburgh as in a previous study with a CBG-AAT chimera [18]. Thus, CBG displays the wild-type sequence in all functionally important areas. In the crystal structure the peptide chain could be traced contiguously in the electron density maps except for two regions (Fig. 1). First, the loop that interconnects helix D and strand 2 of β-sheet A is not resolved. This is in contrast to previous structures where the loop was visible at least in some of the molecules in the asymmetric unit [17], [18]. Second, the RCL was cleaved during crystallization, and upon structure solution it became obvious that CBG is depicted in the SERPIN R-conformation with a fully antiparallel six-stranded β-sheet A, in which the RCL is integrated as β-strand s4 into sheet A [37].

**Figure 2:**
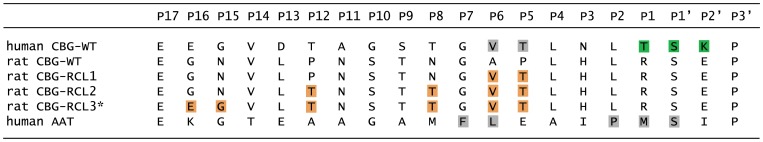
Alignment of reactive center loop sequences of CBG variants and of closely related AAT. The RCL sequence shows a high degree of variance even within closely related proteins. The numbering of residues in the RCL of SERPINs is commonly in relation to the normal cleavage site in AAT between P1 and P1′. Already known cleavage sites are shown with grey boxes. The new cleavage site in human CBG observed in the structure is depicted in green. Introduced mutations in rat CBG-RCL1 through RCL3 are marked by orange boxes. The variant marked with an asterix bears in addition two mutations in the top of β-sheet A deleting a salt-bridge by converting it to amino acids present in human CBG (D323N, R174K). The wild-type protein sequences are annotated in the Uniprot knowledgebase with the following accession codes: rat CBG, P31211; human CBG, P08185; human AAT; P01009. The amino acids in the RCL are numbered following the convention of Schechter and Berger, 1967 [49].

**Figure 3:**
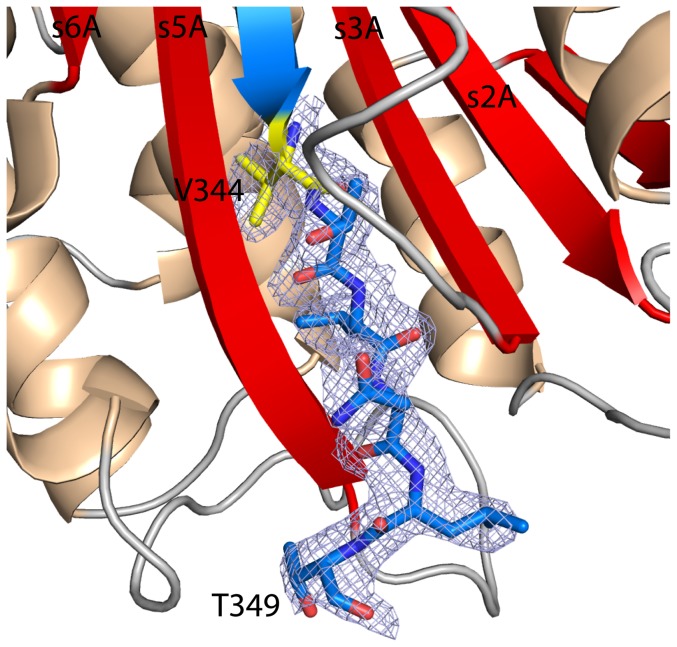
Insertion of the cleaved RCL into β-sheet A reveals a novel cleavage site. The cleavage site utilized by neutrophil elastase is C-terminal of Val344 (yellow). The σ_A_-weighted 2*F_o_-F_c_* electron density map contoured at 1 σ is displayed around residues 344 to 349, showing clearly the completion of β-sheet A. This elongation triggered by a different cleavage site leads to the formation of 6 additional hydrogen bonds. Residue Ser350 is not visible in the electron density maps. It is either part of this segment or of the new N-terminus.

It is not clear why CBG was cleaved during crystallization, but the cleavage site differs from the previously determined neutrophil elastase cleavage site in human CBG (Fig. 2) [5]. Inspection of the electron density maps narrows down the exact cleavage position to two residues (Fig. 3). The RCL is cleaved after Thr349 or Ser350. Residues Thr349 and Lys351 are well defined in the electron density maps and are located before and after the chain break, while unambiguous density is missing for residue Ser350. If cleavage occurred after Thr349, the cleavage site would be homologous to that of human AAT after cleavage with neutrophil elastase (Fig. 2). As a result of this novel cleavage site, the inserted RCL segment is four residues longer than expected, and a more extended β-strand s4 is formed with additional main-chain hydrogen bonds with neighboring strands s3 and s5 (Fig. 3).

The overall RMSD between the Cα chain trace of the CBG structure presented here and the CBG-AAT chimera structure (PDB id 2VDY) is as low as 0.75 Å (Supplemental Fig. S1). This low value shows that neither the non-natural RCL that is present in the CBG-AAT chimera structure nor the human CBG single point mutation K126A studied here substantially alters the overall structure of CBG [18]. The latter was anticipated because residue 126 is located on the surface and is remote from the steroid-binding site and the RCL insertion site.

### Progesterone-binding determinants in human CBG

**Figure 4:**
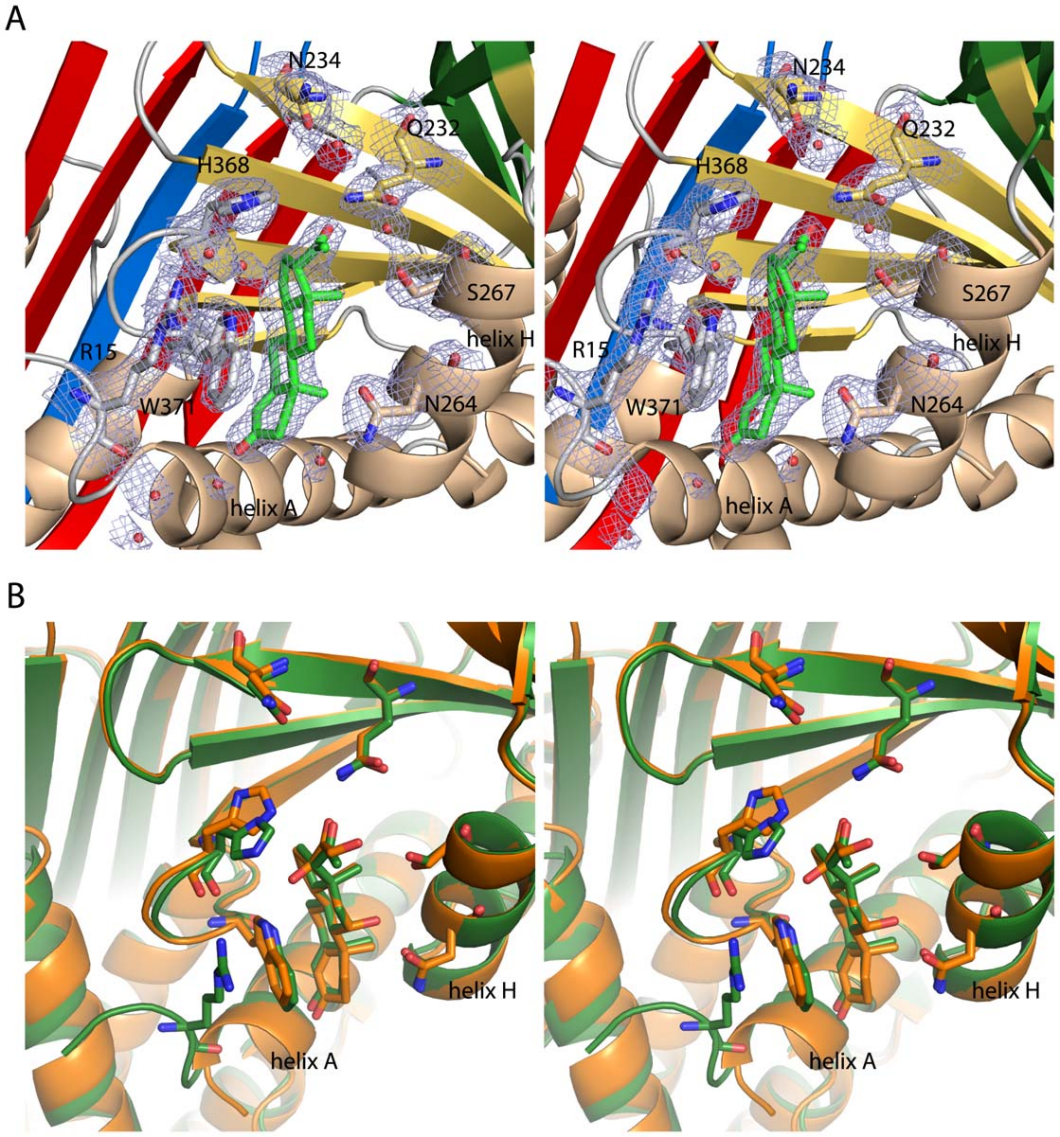
Stereo-views of the hormone-binding pocket. (**A**) Coordination of progesterone in the binding site. Key residues for hormone binding are shown as stick representation. Water molecules surrounding the binding site are depicted as red dots. The σ_A_-weighted 2*F_o_-F_c_* electron density map contoured at 1 σ is shown for the depicted amino acids, ligand and water molecules. The cation-π-stacking of R15 with W371, which has also been seen in the rat CBG structure [17], is well defined in the density. (**B**) Comparison of the binding pocket between cleaved human CBG in complex with progesterone and cleaved human CBG soaked with cortisol. The structures were aligned by the Cα chains of strand 4 and 5 of β-sheet B and helix H (residues 363–380 and 261–268, respectively). They show a very high degree of congruence, but differ slightly in the orientation of side chains which can form additional hydrogen bonds in the case of the ligand cortisol.

Although the structure adopts the conformational R-state, and hence the predicted low affinity conformation [5], [38], inspection of the electron density maps shows unambiguous density for the steroid in the binding pocket (Fig. 4*A*). Progesterone is bound on top of β-sheet B in an orientation that is identical to that of cortisol in previous CBG structures (Fig. 4*B*) [17], [18]. The stacking of the indol ring of Trp371 against the cyclohexane ring of progesterone constitutes a hallmark for ligand binding to CBG. Trp371 itself packs via a cation-π-interaction with Arg15. Only a single direct hydrogen bond is formed between progesterone and CBG, namely between the carbonyl oxygen at C-20 and the side chain of Gln232. The two carbonyl oxygen atoms at C-3 and C-20 of progesterone interact with two water molecules thereby allowing for additional bridging hydrogen bonds to be formed with CBG residues.

A similar stacking interaction with the indol ring of Trp371 is also characteristic for the binding of cortisol to rat and human CBG [17], [18]. However, the additional cation-π-interaction of the indol ring with an arginine residue can only be observed in the rat CBG-cortisol complex but not in the human CBG-AAT chimera structure. Cortisol differs from progesterone by three additional hydroxyl groups that are attached to carbon atoms C-11, C-17 and C-21 in the cortisol structure, which allow for additional polar interactions with CBG residues. Thus for example, the hydroxyl group at C-11 forms a hydrogen bond with Asp256 in rat CBG and Asn264 in human CBG, respectively [17], [18]. The C-17 hydroxyl group of cortisol is in hydrogen bond distance to His368 in human CBG. In the rat CBG structure the C-21 hydroxyl group hydrogen-bonds to a water molecule, which is coordinated between Gln224 and Gly259. Clearly, the absence of these hydroxyl groups in the ligand progesterone could easily account for reduced binding affinity of progesterone [39].

### The structural integrity of human and rat CBG variants is confirmed by CD spectroscopy

**Figure 5:**
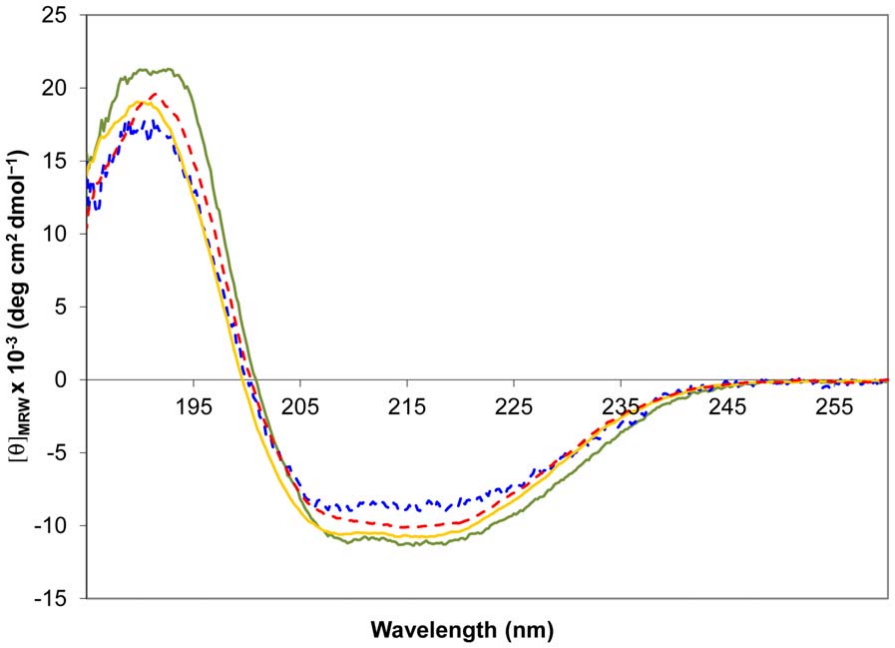
Far-UV CD spectra of CBG. The spectra confirm folded protein before and after cleavage of the RCL. Cleaved samples have been incubated with neutrophil elastase prior to buffer exchange. Native rat CBG-WT (green line) and cleaved rat CBG-RCL1 (blue broken line) show a similar curve shape with slight difference in signal strength, which is most probably due to errors in concentration measurements. In the comparison of native human CBG (yellow line) and cleaved human CBG (red broken line) a slight change in curve shape in the area around 208 nm is visible.

In addition to the single site human CBG mutant K126A (see above) we analyzed rat CBG-WT, CBG-RCL1 (A335V, P336T), CBG-RCL2 (variant CBG-RCL1 plus mutations P329T and N333T) and CBG-RCL3 (CBG-RCL2 plus R174K, D323N, G325E and N326G) in order to compare species-specific structure-function implications during RCL cleavage (Table S1 and Fig. 2). In rat CBG-RCL1 two residues are mutated in the RCL in order to obtain a protein that can be cleaved with human neutrophil elastase. In CBG-RCL2 and CBG-RCL3 additional residues are substituted against the corresponding human residues. Except for rat CBG-WT, which cannot be cleaved by the human enzyme, similar amounts of human neutrophil elastase are required to cleave all the variants. Cleavage results in an approximately 4.5 kDa shift on a SDS-PAGE gel and can be followed to completion (Supplemental Fig. S2). Uncleaved and cleaved samples of rat and human CBG display far-UV CD spectra that are characteristic for natively folded proteins (Fig. 5, Supplemental Fig. S3).

### Steroid-binding affinities monitored by isothermal titration calorimetry

**Figure 6:**
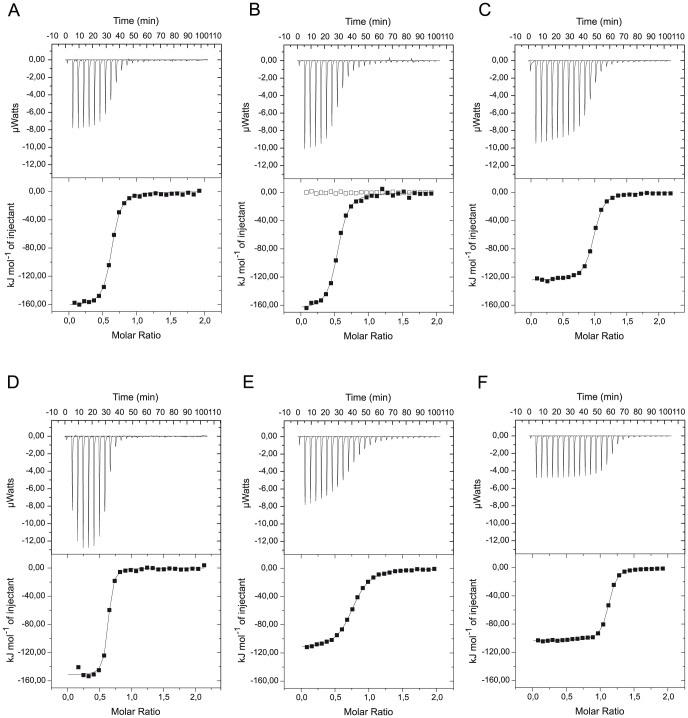
Isothermal titration calorimetry measurements of steroid binding to rat and human CBG. (A–F) Binding isotherms of titrations with hydrocortisone and corticosterone. (A) uncleaved rat CBG-RCL1 titrated with hydrocortisone (B) cleaved rat CBG-RCL1 titrated with hydrocortisone (C) uncleaved rat CBG-WT titrated with hydrocortisone (D) uncleaved human CBG-WT titrated with hydrocortisone (E) cleaved human CBG-WT titrated with hydrocortisone (F) uncleaved rat CBG-WT titrated with corticosterone. A reference titration with hydrocortisone in the absence of protein is shown as open dots in panel B.

Titration experiments were performed to determine the steroid-binding affinities of rat and human CBG before and after cleavage of the RCL. Titrations with cortisol and corticosterone resulted in two-state binding curves, and the binding characteristics could be readily determined (Fig. 6, Table 2). Deviations from the expected 1∶1 ratio of protein and ligand, as indicated from the inflection point shift in some measurements, likely result from sample impurities and inaccuracies in concentration determinations. Our measurements show the same tendency in the thermodynamic parameters Δ*H*° and *T*Δ*S*° as previously observed in equilibrium dialysis experiments [39]. Neutrophil elastase cleavage of human CBG results in a change in *K*_d_ from 45 nM prior cleavage to 442 nM post cleavage. In contrast, the RCL cleavage in rat CBG leads to a smaller, about 2-fold, decrease in binding affinity. The *K*_d_ value increases from 95 and 122 nM in the uncleaved proteins rat CBG-RCL1 and rat CBG-WT, respectively, to 209 nM post cleavage in rat CBG-RCL1 (Table 2). The observed ten-fold reduction in human CBG upon RCL cleavage compares well to that previously reported [5], [22].

### Differences in the thermal denaturation behavior between human and rat CBG

**Figure 7:**
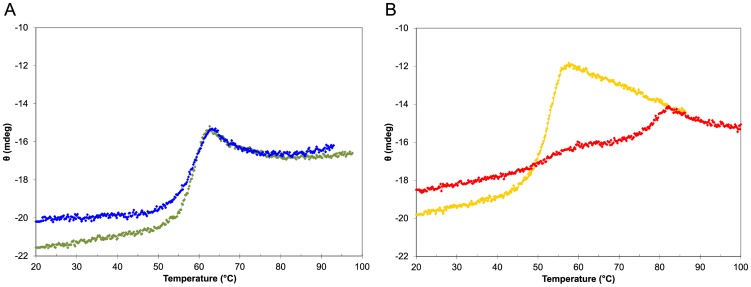
Thermal denaturation monitored by CD spectroscopy. Measurement at a single wavelength while increasing temperature reveals the high thermal stability of cleaved human CBG. (**A**) Rat CBG shows no difference in thermal stability upon cleavage of the RCL. Native rat CBG-WT is shown in green, cleaved rat CBG-RCL1 in blue. The shape of the curves is largely similar. (**B**) Human CBG shows great differences in thermal stability between cleaved and native protein. The cleaved state of human CBG is highly thermostable (red), while the native state (yellow) shows a curve similar to rat CBG.

Human CBG behaves as anticipated in a CD-monitored thermal denaturation experiment. Uncleaved human CBG-WT unfolds at approximately 55°C, while the neutrophil elastase-cleaved protein is considerably more stable up to around 80°C, as previously noted [5] (Fig. 7*B*). This increase in thermal stability is a well-recognized property of SERPINs that undergo an S-to-R transition [35]. Conversely, no increase in thermal stability is observed when such a transition does not take place, irrespectively of whether the RCL loop is cleaved or not [40], [41]. Surprisingly, this latter behavior is observed in uncleaved rat CBG-WT and cleaved rat CBG-RCL1. Both proteins behave similarly upon heating and unfold at around 58°C which indicates that the S-to-R transition does not occur in rat CBG after cleavage with human neutrophil elastase (Fig. 7*A*). To validate the experimental setup, we repeated the experiment using identical conditions with a cleaved and uncleaved sample of human alpha-1-antitrypsin (Supplemental Figs. S3 and S4). As expected for this archetypical member of the SERPIN family [42], we observe a behavior that is similar to human CBG, namely an increase in thermal stability from about 60°C to higher than 90°C concomitant with the cleavage of the RCL.

The absence of an S-to-R transition in rat CBG is further supported by native gel electrophoresis experiments (Supplemental Fig. S2). If analyzed under similar conditions, uncleaved and elastase-cleaved rat CBG-RCL1, as well as uncleaved human CBG-WT, show a highly similar tendency to aggregate after a short-time exposure to 70°C. In contrast, elastase-cleaved human CBG-WT does not appear to aggregate upon heating, in agreement with the expected higher thermal stability of the conformational R-state. Conversely, the conformational R-state appears not to be accessible to rat CBG since no difference can be observed between elastase-cleaved and uncleaved CBG-RCL1.

In order to investigate whether individual substitutions in the RCL are responsible for the differences in the behavior of human and rat CBG, we introduced additional substitutions in rat CBG mutants CBG-RCL2 and CBG-RCL3 (Fig. 2). In rat CBG-RCL2 we substituted an asparagine residue at position P8 and a proline at position P12 against two threonine residues found at these positions in human CBG. While a threonine residue at P8 might be better accommodated in general in the R-conformation, the residue at position P12 possibly has a more specific role since it becomes part of the so-called SERPIN shutter upon RCL insertion [4], and it has been proposed that a non-proline residue at position P12 is required for the successful insertion of the RCL [37], [38]. In rat CBG-RCL3, we switched residues P15 and P16 to the human residues and also deleted a salt bridge that forms between β-strands 3 and 5 in β-sheet A, which could possibly hinder insertion of the RCL. This salt bridge is a feature of rat but not human CBG. The purified elastase-cleaved and uncleaved rat CBG-RCL2 and CBG-RCL3 displayed a CD spectra characteristic for folded proteins (data not shown for CBG-RCL2; Supplemental Fig. S3). However, neither of these humanized rat CBG variants displayed an increased thermal stability upon RCL cleavage (data not shown for CBG-RCL2; Supplemental Fig. S4).

## Discussion

The identification of a novel proteolytic cleavage site in the RCL of human CBG was unexpected. If one assumes that cleavage occurs after Thr349 (instead of Ser350, see above) then this would be identical to the P1-P1′ cleavage site in AAT and clearly different from the human elastase cleavage site in human CBG positioned between P7 and P6 when following the AAT numbering (Fig. 2). From a structural point of view, this novel cleavage site makes sense because it allows for the formation of an extended β-strand s4A with main-chain hydrogen-bonds connecting the former RCL segment to strands s5A and s3A over the entire length of β-sheet A (Fig. 3). In contrast, the previously identified cleavage site after residue Val344 in human CBG would give rise to a strand s4A that is shorter by three residues in comparison to the neighboring strands s5A and s3A, and this would bury a C-terminal carboxylate group in a partially hydrophobic environment [5].

This novel protease cleavage site offers the perspective that yet unknown proteases might exist, which are able to trigger the S-to-R transition in human CBG. These proteases might be species-specific since the CBG RCL sequences vary considerably between species [1]. In fact, this property extends to the entire SERPIN family where the RCL segments share the lowest sequence identities among all stretches between different SERPINs and even between the same SERPINs in different species [37].

Structural investigations reported for rat and human CBG as well as human TBG were performed with proteins produced in *E. coli* [17], [18], [19], [22]. These studies do not take into account that human CBG displays a potential N-glycosylation site at position P3 of the RCL, namely at residue Asn347. Recently, it has been shown that this site is occupied in over 80% of CBG molecules from human serum [3]. If human CBG is cleaved at the human elastase cleavage site after the residue P7, then residue Asn347 will not become part of the inserted RCL segment. In contrast, if cleavage occurs at the site seen in the crystal structure, the glycosylated Asn347 would be part of the inserting RCL and needs to be accommodated at the end of strand s4A of CBG. Although the side chain of Asn347 appears to be partially accessible from the surface in the present crystal structure, we cannot rule out that glycosylation at this site will affect the overall structure of CBG and its steroid-binding properties. Furthermore, we cannot rule out that glycosylation influences the accessibility of the cleavage site to proteases in human and rat CBG.

A comparison of the positioning of progesterone and cortisol in the CBG steroid-binding site shows that both steroids bind in the same orientation raising the question of which residues are responsible for the reduced affinity for progesterone (Fig. 4*B*). Available binding data from fluorescence measurements state a *K*_d_ of approximately 85 nM for binding of progesterone to human CBG at 37°C compared to 18 nM for cortisol [21]. Our structural data suggest that this difference can be attributed to the fact that progesterone does not form some of the hydrogen bonds observed in the cortisol complex because it lacks the hydroxyl groups attached to atoms C-11 and C-17. The absence of an hydroxyl group at C-21 in the steroid side chain of progesterone does not seem to contribute to its lower binding affinity because this hydroxyl group does not participate in any defined interactions in the cortisol complex of human CBG (PDB id 2VDY) [18].

The extent to which the S-to-R transition in human CBG affects steroid-binding affinity has been the subject of debate [1], [22], but this can be explained by differences in the assays used, the assay conditions, and the materials analyzed. Saturation ligand-binding assays have been widely employed for this purpose, and rely on radiolabeled steroids to measure their binding kinetics *via* Scatchard analysis [43]. Some of these assays, and particularly those that involve the use of dextran-coated charcoal to separate bound from free steroid ligands, are sensitive to perturbations in binding affinity, and will not reliably detect interactions that fall below an affinity (*K*_d_) of ∼10 nM [44]. Furthermore, these types of assays are conducted at ∼0°C to minimize the stripping of radio-ligands from the steroid-binding sites of proteins. This is important because steroid-binding kinetics are strongly influenced by temperature. The sample type will also influence Scatchard analyses because typically only the bound radioligand is measured and the actual amount of free ligand may be highly influenced by the presence of other steroid-binding proteins in the sample, such as albumin. It is therefore difficult to directly compare affinity constants obtained using different assays and sample types, and especially between glycosylated CBG produced in mammalian cells with unglycosylated CBG produced by *E. coli* since we previously observed that the presence of specific carbohydrate chains on CBG influences the formation of a high-affinity cortisol-binding site in proteins expressed in mammalian cells [45]. In the present study we used ITC measurements at 25°C in combination with *E. coli* produced protein and obtained affinities of 45 and 442 nM for binding of cortisol to uncleaved and elastase-cleaved human CBG, respectively. These values compare well with those from fluorescence titration data that showed that uncleaved human CBG displays a binding affinity of 26.9 nM for cortisol at 22°C and that the affinities of uncleaved and cleaved human CBG differ by about 4.7-fold at 37°C [22].

Human CBG behaves as expected for SERPINs that undergo an S-to-R transition, and our thermal denaturation and structural studies on human CBG fully agree with previous characterizations [5], [18], [38]. SERPIN-typical S-to-R transition behavior can also be readily reproduced with human AAT (Supplemental Fig. S4B) [19], [42], [46]. However, in the case of rat CBG the S-to-R transition does not take place, and this is also supported by the observed ligand-binding affinities. Whereas in human CBG we observe a change in the ligand-binding affinity of about ten-fold between the uncleaved and cleaved proteins, while in rat CBG the binding affinity only changes about two-fold. Whether this small reduction in the steroid-binding affinity of rat CBG after cleavage of the RCL is sufficient to exert the same effects on the plasma distribution of steroids, as observed after human CBG is cleaved by neutrophil elastase remains to be determined [47].

While the mutation of a single residue in human CBG appears to block the S-to-R transition [38], we have not been able to pinpoint the difference in the S-to-R transition behavior between human and rat CBG to individual rat CBG residues. Introduction of multiple mutations in the RCL of rat CBG did not yield any indication for the formation of the R-conformation (Fig. 2). This was also true for mutant RCL3, where in addition to substitutions in the RCL we also targeted a salt bridge that is formed between β-strands s3A and s5A in β-sheet A in rat CBG and, which we hypothesized might therefore block insertion of the RCL segment between the β-strands in sheet A. It remains to be seen whether the S-to-R transition can be triggered in rat CBG through the introduction of single key amino acid substitutions as has previously been achieved for ovalbumin [41], [48].

Our observation that human CBG undergoes an S-to-R transition whereas this transition is absent in rat CBG cautions against the common practice of deriving general conclusions from the comparison of the conformational states of proteins from different species. The “flip-flop” mechanism proposed by us and others for the reversible binding of steroids to CBG assumes that the RCL has an inherent tendency to insert between strands 3 and 5 of β-sheet A even in uncleaved CBG. The “flip-flop” mechanism posits that the RCL is partially inserted into sheet A when no ligand is present and becomes fully exposed upon ligand binding. This mechanism was derived from a compilation of structural data from uncleaved rat CBG, cleaved human CBG and uncleaved human TBG. The unsuspected differences between rat and human CBG revealed in the present study question whether such a common mechanism exists for these proteins. Clearly, additional structures, such as for example crystal structures of uncleaved rat and human CBG with no steroid bound, are needed in order to derive a better understanding for how the structure enables function in CBG.

## Supporting Information

Figure S1 **Superposition of cleaved human CBG-WT (green) with the cleaved human CBG-AAT chimera from Zhou et al. (PDB id 2VDY)(orange).** The alignment was based on the Cα chains of residues 16 to 383. (**A**) Overlay with chain A from 2VDY (RMSD 0.763 Å). (**B**) Overlay with chain B from 2VDY (RMSD 0.714 Å).
(TIF)

Figure S2 **Characterization of a rat and human CBG.** (A) SDS-PAGE of samples used for the native PAGE shown in panel (B). (**B**) Native PAGE (7.5%) of uncleaved (uc) and cleaved (c) samples before and after a 70°C heating step. Clear differences can be seen between rat and human CBG: Elastase cleavage of human CBG yields a thermostable protein which is considerably less prone to aggregation than uncleaved human CBG and cleaved and uncleaved rat CBG. This is consistent with the observation that human CBG undergoes an S-to-R transition upon cleavage whereas rat CBG does not. The heating step was carried out as follows: A 12 µM protein solution in 10 mM potassium phosphate pH 7.4 was heated to 70°C at 0.1°C/s heating rate in a thermocycler. The temperature was kept at 70°C for 180 sec before cooling to 4°C. The electrophoretic separation was performed at 4°C.
(TIF)

Figure S3 **Far-UV CD spectra of rat CBG-RCL3 and human AAT.** The shape of the rat CBG (black line) curve is comparable to that of other CBG variants. Cleaved RCL3 is shown as ocher broken line. Uncleaved and cleaved human AATs are depicted as blue and ruby broken lines, respectively.
(TIF)

Figure S4 **Thermal denaturation monitored by CD spectroscopy.** The thermal denaturation of rat CBG-RCL3 and human AAT highlights the increased stability of human AAT after RCL cleavage which does not occur in CBG-RCL3. (**A**) The rat CBG-RCL3 variant shows no increase in stability after elastase cleavage (uncleaved CBG-RCL3 in red, cleaved CBG-RCL3 in green). (**B**) While native AAT (in blue) shows a denaturation profile similar to native CBG, cleaved AAT (in purple) is considerably more stable up to 90°C.
(TIF)

Table S1 **Primer sequences for site directed mutagenesis.**
(DOCX)
