# Supplementary figures and images for: Corticosteroid-Binding Globulin: Structure-Function Implications from Species Differences
Source: PLoS One. 2012 Dec 26;7(12):e52759. doi: 10.1371/journal.pone.0052759 (PMC3530532; doi:10.1371/journal.pone.0052759)

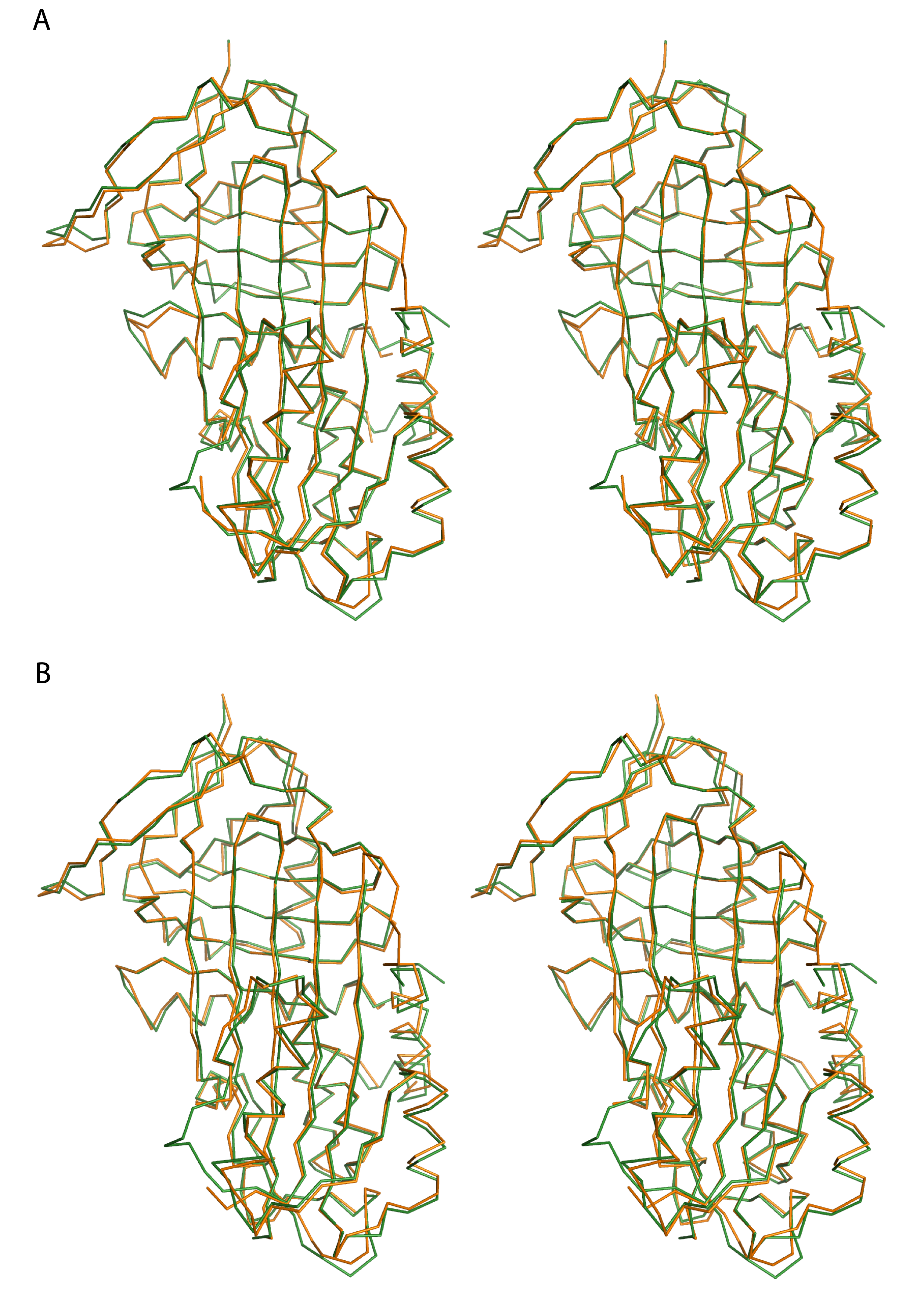

Supplement: Figure S1 — Superposition of cleaved human CBG-WT (green) with the cleaved human CBG-AAT chimera from Zhou et al. (PDB id 2VDY)(orange). The alignment was based on the Cα chains of residues 16 to 383. (A) Overlay with chain A from 2VDY (RMSD 0.763 Å). (B) Overlay with chain B from 2VDY (RMSD 0.714 Å). (TIF) [file pone.0052759.s001.tif]

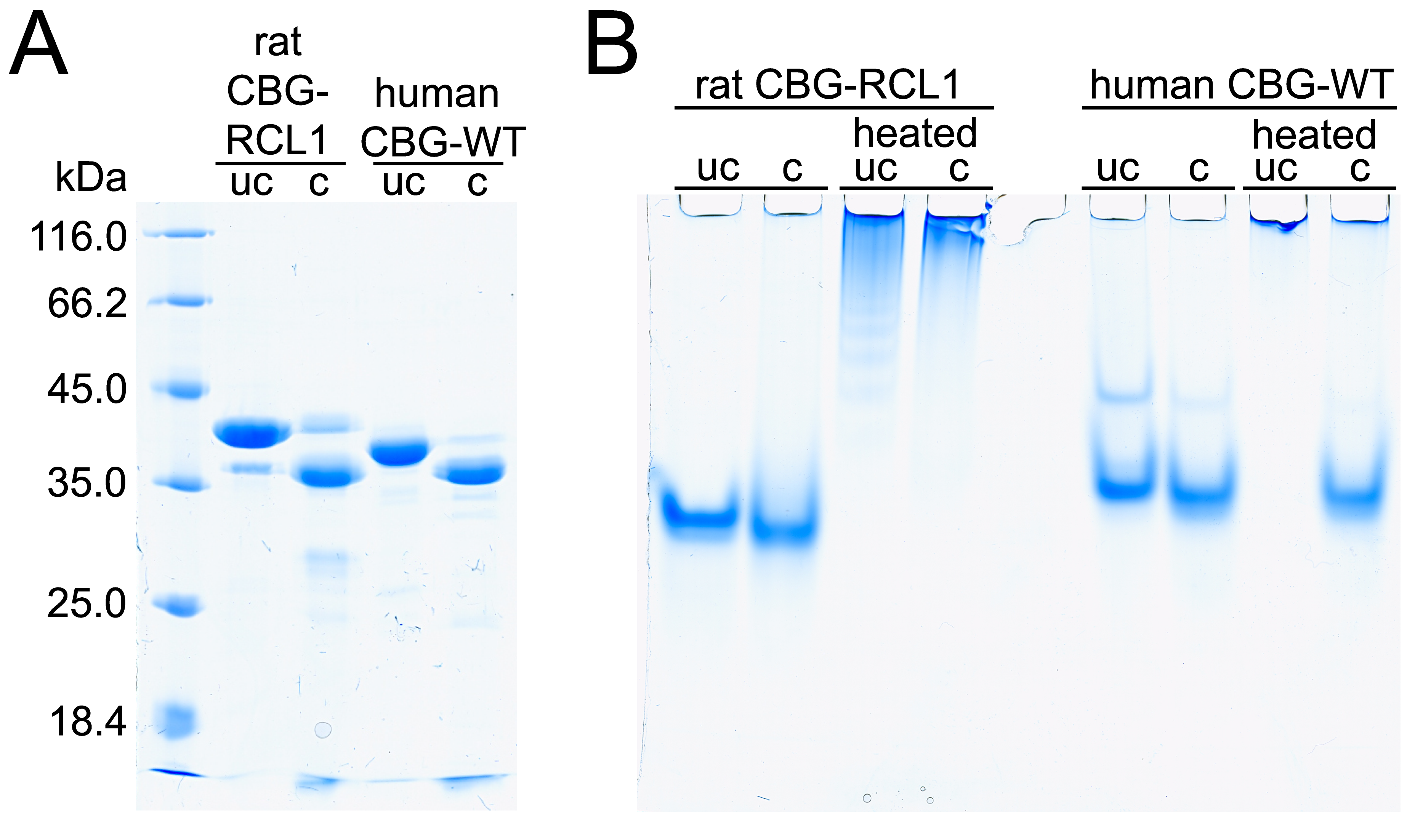

Supplement: Figure S2 — Characterization of a rat and human CBG. (A) SDS-PAGE of samples used for the native PAGE shown in panel (B). (B) Native PAGE (7.5%) of uncleaved (uc) and cleaved (c) samples before and after a 70°C heating step. Clear differences can be seen between rat and human CBG: Elastase cleavage of human CBG yields a thermostable protein which is considerably less prone to aggregation than uncleaved human CBG and cleaved and uncleaved rat CBG. This is consistent with the observation that human CBG undergoes an S-to-R transition upon cleavage whereas rat CBG does not. The heating step was carried out as follows: A 12 µM protein solution in 10 mM potassium phosphate pH 7.4 was heated to 70°C at 0.1°C/s heating rate in a thermocycler. The temperature was kept at 70°C for 180 sec before cooling to 4°C. The electrophoretic separation was performed at 4°C. (TIF) [file pone.0052759.s002.tif]

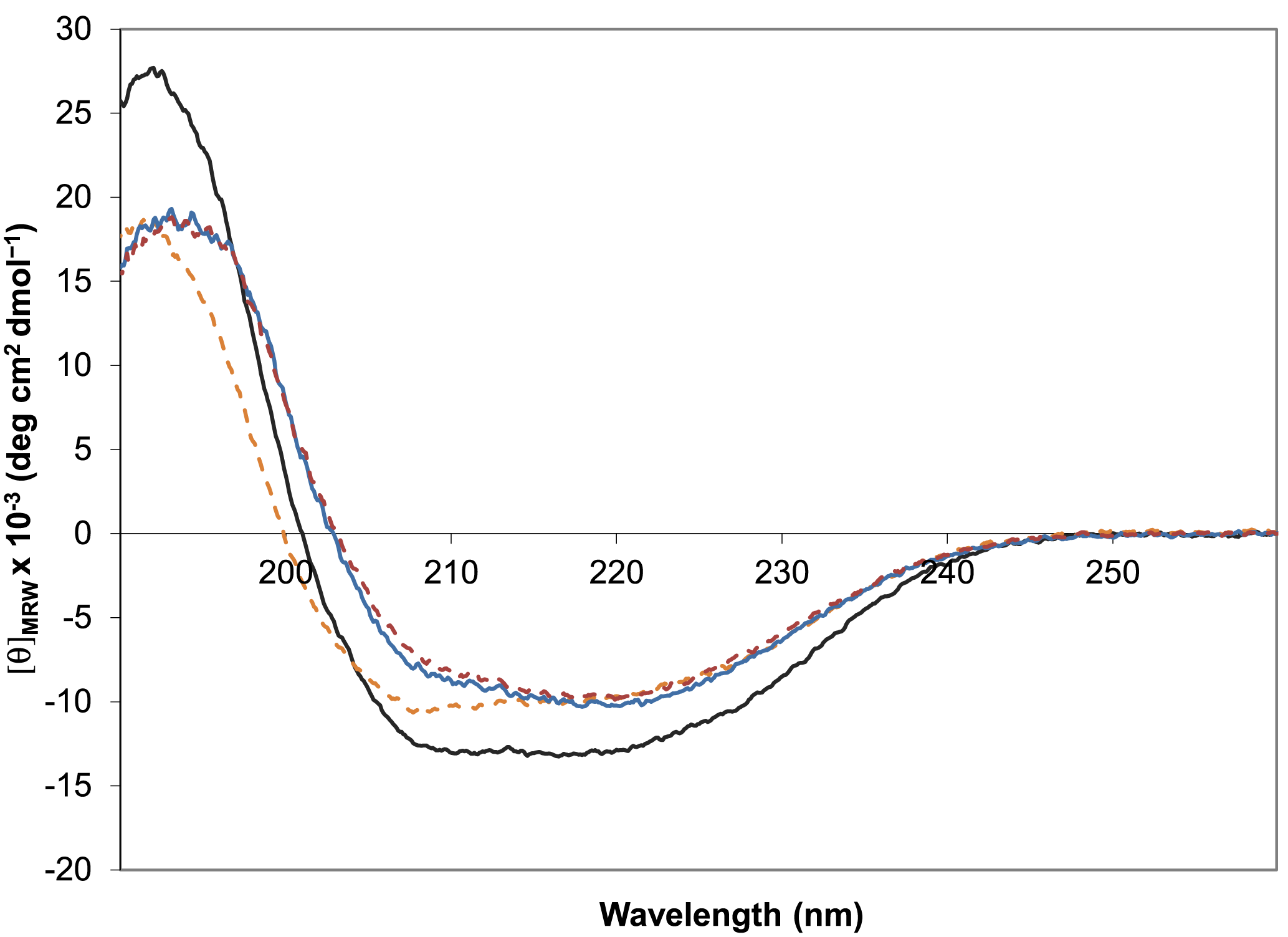

Supplement: Figure S3 — Far-UV CD spectra of rat CBG-RCL3 and human AAT. The shape of the rat CBG (black line) curve is comparable to that of other CBG variants. Cleaved RCL3 is shown as ocher broken line. Uncleaved and cleaved human AATs are depicted as blue and ruby broken lines, respectively. (TIF) [file pone.0052759.s003.tif]

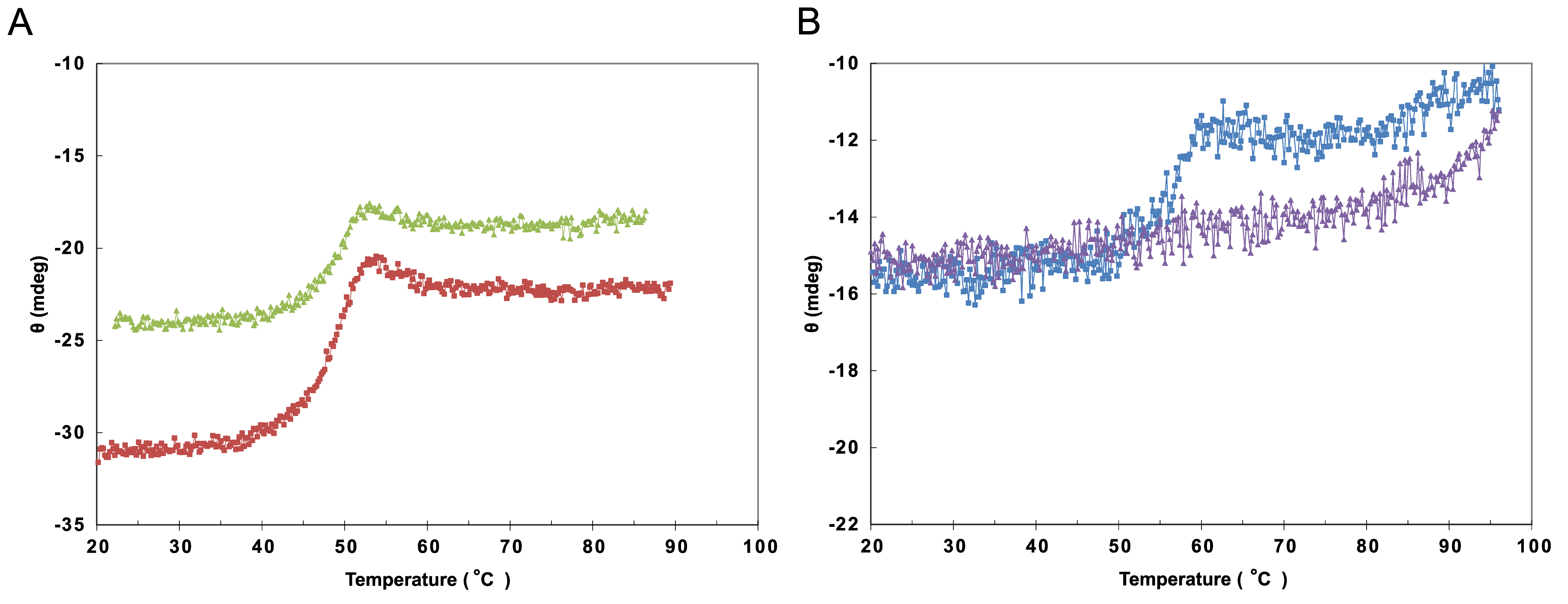

Supplement: Figure S4 — Thermal denaturation monitored by CD spectroscopy. The thermal denaturation of rat CBG-RCL3 and human AAT highlights the increased stability of human AAT after RCL cleavage which does not occur in CBG-RCL3. (A) The rat CBG-RCL3 variant shows no increase in stability after elastase cleavage (uncleaved CBG-RCL3 in red, cleaved CBG-RCL3 in green). (B) While native AAT (in blue) shows a denaturation profile similar to native CBG, cleaved AAT (in purple) is considerably more stable up to 90°C. (TIF) [file pone.0052759.s004.tif]
