# Supplementary material for: Corticosteroid-Binding Globulin: Structure-Function Implications from Species Differences
Source: PLoS One. 2012 Dec 26;7(12):e52759. doi: 10.1371/journal.pone.0052759 (PMC3530532; doi:10.1371/journal.pone.0052759)
Supplement: Table S1 — Primer sequences for site directed mutagenesis. (DOCX) [file pone.0052759.s005.docx]

**Table S1**

**Primer sequences for site-directed mutagenesis**

| Construct | Direction | Mutation | Sequence |
| --- | --- | --- | --- |
| Rat CBG-RCL1 | forward | A335V P336T | 5’-CTA ATT CTA CCA ACG GGG TTA CCC TAC ACC TGC GCT CTG-3’ |
|  | reverse | A335V P336T | 5’-CAG AGC GCA GGT GTAGGG TAA CCC CGT TGGTAG AAT TAG-3’ |
| Rat CBG-RCL2 | forward | N333T A335V P336T | 5’-GCC TAA TTC TAC CAC CGG GGT GAC CCT ACA CCT GCG C-3’ |
|  | forward | P329T | 5’-GAT GAA GGG AAT GTG TTG ACC AAT TCT ACC ACC GGG GTG-3’ |
|  | reverse | N333T A335V P336T | 5’-GCG CAG GTG TAG GGT CAC CCC GGT GGT AGA ATT AGG C-3’ |
|  | reverse | P329T | 5’-CAC CCC GGT GGT AGA ATT GGT CAA CAC ATT CCC TTC ATC-3’ |
| Rat CBG-RCL3 | forward | R174K | 5'-CCT GGT CAA CTA CAT CTT CCT CAA AGG CAT ATG GGA-3' |
|  | forward | D323N | 5'-AGG CCA TGC TAC AAC TGA ATG AAG AAG GTG TGT TG-3' |
|  | forward | G325E N326G | 5’-GGC CAT GCT ACA ACT GGA TGA AGA AGG TGT GTT GAC CAA TTC TAC CAC CG-3’ |
|  | forward | P329T | For primer sequence see rat CBG-RCL2 |
|  | forward | N333T A335V P336T | For primer sequence see rat CBG-RCL2 |
|  | reverse | R174K | 5'-TCC CAT ATG CCT TTG AGG AAG ATG TAG TTG ACC AGG-3' |
|  | reverse | D323N | 5'-CAA CAC ACC TTC TTC ATT CAG TTG TAG CAT GGC CT-3' |
|  | reverse | G325E N326G | 5’-CGG TGG TAG AAT TGG TCA ACA CAC CTT CTT CAT CCA GTT GTA GCA TGG CC-3’ |
|  | reverse | P329T | For primer sequence see rat CBG-RCL2 |
|  | reverse | N333T A335V P336T | For primer sequence see rat CBG-RCL2 |
| Human CBG | forward | K126A | 5’-GTC ATT CTC AGC AGA CAT CGC GCA CTA CTA TGA GTC AGA G-3’ |
|  | reverse | K126A | 5’-CTC TGA CTC ATA GTA GTG CGC GAT GTC TGC TGA GAA TGA C-3’ |
